# Experimental Atherosclerosis Research on Large and Small Animal Models in Vascular Surgery

**DOI:** 10.1159/000524795

**Published:** 2022-06-27

**Authors:** Florian Simon, Axel Larena-Avellaneda, Sabine Wipper

**Affiliations:** ^a^University Hospital Düsseldorf, Clinic for Vascular and Endovascular Surgery, Heinrich-Heine-University Düsseldorf, Düsseldorf, Germany; ^b^Department of Vascular and Endovascular Surgery, Asklepios Clinic Altona, Hamburg, Germany; ^c^Department for Vascular Surgery, University Hospital Innsbruck, Innsbruck, Austria

**Keywords:** Animal model, Atherosclerosis, Transgenic models, ApoE knockout, High-density lipoprotein, Low-density lipoprotein

## Abstract

Animal models have significantly advanced our understanding of the mechanisms of atherosclerosis formation and the evaluation of therapeutic options. The current focus of research is on preventive strategies and includes pharmacologic and biologic interventions directed primarily against smooth-muscle cell proliferation, endovascular devices for recanalization and/or drug delivery, and an integrated approach using both devices and pharmacobiologic agents. The experience over many decades with animal models in vascular research has established that a single, ideal, naturally available model for atherosclerosis does not exist. The spectrum ranges from large animals such as pigs to small animal experiments with genetically modified rodents such as the ApoE−/− mouse with correspondingly differently pronounced changes in their lipid and lipoprotein levels. The development of transgenic variants of currently available models, e.g., an ApoE-deficient rabbit line, has widened our options. Nevertheless, an appreciation of the individual features of natural or stimulated disease in each species is of importance for the proper design and execution of relevant experiments.

## Introduction

In the field of medical research, animal models in which human disease patterns or anatomical and pathophysiological models can be simulated and examined are invaluable. Already in the application for approval for an animal study, the authorities therefore enquire the criteria on which the selection of an experimental set-up is based. Animal models not only make it possible to better understand and analyse the pathomechanism of a disease but also are suitable for the evaluation of therapeutic measures within the framework of preclinical studies. When establishing new drugs, surgical techniques, or medical devices, preclinical testing of safety and efficacy in a model that can be transferred to humans is indispensable. About one-third of highly published and well-cited animal studies are successfully translated into controlled randomized clinical trials [1]. There are a large number of different animal models, from small to large animals, which can be used according to the respective question to be investigated. Research into chronic diseases of the vascular system, such as arteriosclerosis, is also dependent on animal models. The reason for conducting basic research on animals in vascular medicine is that humans have already reached a very advanced stage of arteriosclerosis by the time they become patients. However, research is not only concerned with the final stage of a disease but should also keep an eye on the preliminary stages and even prophylaxis. Animal models offer the possibility to investigate these questions through targeted questions and appropriately provoked disease stages of arteriosclerosis.

Atherosclerosis is a complex, multifactorial disease which, in addition to the primary cardiovascular risk factors (arterial hypertension, hyperlipidaemia, nicotine abuse, obesity, and diabetes mellitus), may also have genetic and inflammatory causes. However, these factors do not stand alone but are mutually dependent. The named primary cardiovascular risk factors are also interrelated to a dysregulated inflammation reaction and/or inflammatory response. It is a complex, chronic inflammatory disease that, depending on the predisposing factors, can affect both the small, capillary vessels and the large conducting vessels [2, 3].

- There is no perfect animal model

An ideal animal model should be as close as possible to humans in terms of anatomy, physiology, and biochemical metabolic properties, including lipid metabolism. Plaque characteristics including pathogenesis, topography, and composition should also be as close as possible to the “patient,” and complications such as plaque rupture and acute vessel occlusion should be simulatable. For experimental atherosclerosis research, it would be desirable if an animal model were as readily available as possible, easy to handle and use, and inexpensive to keep. The animals should be available in genetically pure lines and should be easily reproducible in animal husbandry with as short a gestation period as possible. Furthermore, it should be possible to set the atherosclerotic lesions easily and quickly without inducing further disease. Last but not least, it would be of great importance if a model could produce reproducible results independent of laboratory groups. In the following, the corresponding possibilities and limits of experimental research on small and large animal models with a focus on atherosclerosis development and treatment will be shown.

## Large Animal Models

### Rhesus Monkeys

Non-human primates are not only phylogenetically similar to humans, but also exhibit human nutritional and metabolic behaviour and can develop atherosclerosis as they age when fed diets raising plasma cholesterol to high human levels [4]. Anatomically and physiologically, this animal model is therefore closest to humans. In order to accelerate the development of atherosclerosis, the insulin-producing β-cells of the pancreas can be destroyed in male rhesus monkeys by means of intravenous injection of alloxan. The resulting diabetes mellitus with consecutive atherosclerosis leads to plaques with a focus on coronary arteries and renal arteries [5]. Furthermore, atherosclerosis can be forced by a diet high in fat and cholesterol. Despite these measures, the period of plaque generation is very time-consuming at 5–6 years, which is why this model is not primarily used for experimental analyses [6, 7].

### Swine

Pigs offer the advantage that the cardiovascular system and metabolism are similar to humans and the vascular system can develop atherosclerotic changes. However, these changes only develop after a correspondingly long time, which is why the animals are fully grown by then, which poses great challenges for husbandry and experimental management with up to 300 kg live weight. Even mini breeds bred specifically for experimental purposes reach a weight class of up to 100 kg and higher when fully grown. However, it is possible to generate atherosclerosis in Hanford or Yucatan mini-pigs within 16–18 weeks by a high-cholesterol and/or high-sugar diet at an early stage in order to use these animals for experimental analyses. Although these interventions make it possible to shorten the experimental times, they always involve a change in the physiology, metabolism, and inflammatory reaction, which until then had been very similar to humans, which is why such experimental approaches require greater scope for interpretation [8, 9, 10].

- Large animal models are often time-consuming and cost-intensive

Furthermore, a pig breed with inherited low-density lipoprotein (LDL) hypercholesterolaemia has been described showing spontaneous haemorrhages and plaque ruptures at coronary lesions at 39–54 months of age [11]. This means that the animals have a lower body weight and are better suited for the experimental studies.

### Sheep

In the literature search, the sheep model was mainly used for restenosis and aneurysm research. No studies were found on research into atherosclerosis in the sheep model.

### Cat and Dog

Cats and dogs are carnivores and have a natural resistance to high-fat diets and the development of atherosclerosis. Furthermore, these animals have a distinct coronary collateral network, which provides special protection against myocardial infarction. These species have been used several times for studies in the context of ischaemia-reperfusion research, although the direct transferability to humans must be questioned. Thus, these animal species are rather unsuitable for atherosclerosis research [12, 13, 14].

## Small Animal Models

### Rabbit

One of the first animal species to be used for atherosclerosis research was the rabbit. Ignatowski [15] developed a rabbit model in 1908 using a special diet of meat, milk, and eggs that led to plaque formation in the aorta. Due to the simple and inexpensive husbandry as well as the lipid metabolism relatively similar to humans, rabbits became very popular in atherosclerosis research. This led to a variety of study foci such as drug studies on statins.

**Fig. 1:**
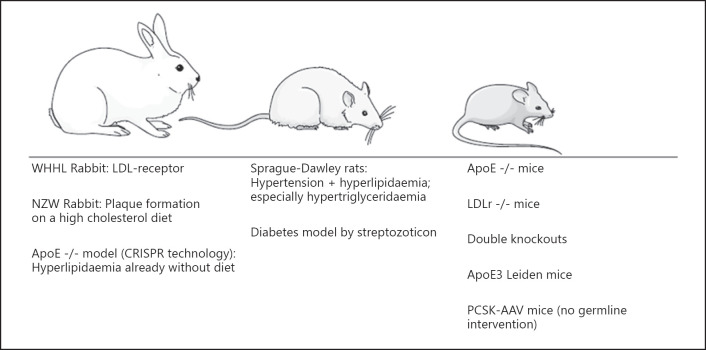
Schematic overview of common small animal models in atherosclerosis research [16]. WHHL, Watanabe heritable hyperlipidaemic.

One of the oldest experimental models is the Watanabe heritable hyperlipidaemic rabbit (shown in Fig. 1). These animals have a defect in the LDL receptor, which is why homozygous rabbits show stably elevated plasma cholesterol levels as early as birth without requiring a special diet. One advantage of this breeding is that the predominant lipoprotein in the plasma is LDL, and the foam cells that develop originate in the vascular smooth muscle cells [17]. The lesions that occur show a differentiated expression and range from fatty deposits in the vessel wall to pronounced lesions of the aorta as well as the coronary and cerebral arteries. In this model, the plaque-stabilizing effect of statins and the effects of insulin resistance on the development of atherosclerosis were investigated [18].

- Diets achieve different results through variable feeding protocols

However, genetic wild-type animals such as the New Zealand White rabbit can also reach extremely high cholesterol levels in a relatively short time within a few weeks by feeding a high-cholesterol diet, but this leads to plaques consisting mainly of foam cells. By feeding a period of several months at lower cholesterol levels, a more differentiated plaque formation can be achieved [19]. Atherosclerotic plaques are not only found primarily in the aortic arch and thoracic aorta but also in the coronaries.

A relatively new method in rabbits to study atherosclerosis is the ApoE−/− model. ApoE is a glycoprotein that is a structural component of all lipoproteins except LDL. The function of ApoE is, among other things, to remove chylomicrons and very low-density lipoprotein (VLDL) remnants as well as to maintain cholesterol homoeostasis [20]. By manipulating the genome, e.g., via CRISPc-associated protein 9 (clustered regularly interspaced short palindromic repeats-associated protein 9), it is possible to breed an ApoE-deficient rabbit line. The advantage compared to the already existing mouse lines with an ApoE deficiency is the lipoprotein profile of rabbits, which is naturally more similar to humans. These animals already show hyperlipidaemia without additional diet, which can be significantly increased using a high-cholesterol diet. The resulting lesions of the aorta are correspondingly more pronounced than in wild type [21]. The Watanabe heritable hyperlipidaemic rabbit type and the ApoE−/− rabbits can thus in combination represent a useful arrangement for the investigation of human hyperlipidaemia, even if the primary localizations of atherosclerosis formation are in the aortic arch and thoracic aorta rather than in the abdominal region.

Although rabbits have been very helpful in understanding the development of atherosclerosis, some drawbacks remain. In order to observe atherosclerotic development and high plasma cholesterol levels in rabbits, a very high-cholesterol diet is necessary, which among other things can lead to liver inflammation and negatively affect the experimental design in this way [22].

### Rat

Rats represent an in-between solution in rodent models as an experimental set-up. On the one hand, they are relatively inexpensive and fast to reproduce. On the other hand, however, they are by far not as widely used and researched as mice, which is reflected in a significantly reduced number of options such as available antibodies or transgenic models. Above all, rats are very resistant to the development of atherosclerotic lesions, which is partly due to the physiologically high levels of high-density lipoproteins and simultaneously low levels of LDL and VLDL in the plasma of the animals [23].

- Rodents are naturally well protected against atherosclerosis

Despite all this, there are some rat models, such as a strain with Sprague-Dawley rats crossed in to study hypertension, but this ultimately resulted in phenotypes suffering from both hypertension and hyperlipidaemia [24]. Another way to generate hyperlipidaemic animals is to feed them a high-fructose diet to increase plasma triglycerides, but this is ultimately only useful for drug trials to study hypertriglyceridaemia and not to observe long-term courses of atherosclerotic plaques. The same applies to trials with i.v. applied tritone derivatives, which cause a tripling of plasma triglyceride and VLDL levels within the first 24 h after administration, but return to normal levels after another 24 h [25]. Another way to establish atherosclerotic plaques is to associate them with a diabetes model. Streptozotocin can be used to induce a diabetic disease in various rodent models, including the rat, which leads to corresponding lesions of the vessels in longer experimental set-ups, but which ultimately always leads to a mixed picture of diseases and can make it difficult to take a closer look at the actual disease pattern [26].

### Mouse

**Fig. 2:**
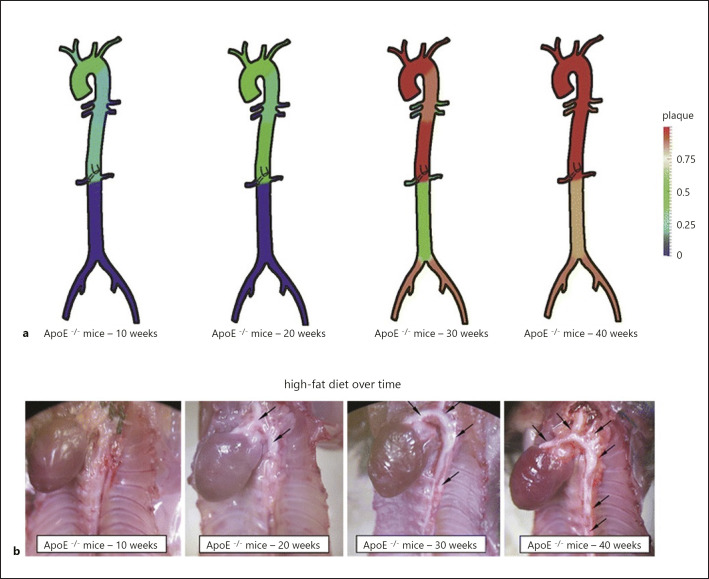
**a, b** Localization of plaque in the ApoE−/− mouse model under high-fat diet over time (10, 20, 30, 40 weeks). **a** Red indicates the presence of plaque, and healthy vessels are coloured blue. **b** Representation in the microscopic image, the arrows point to the regions with the greatest plaque formation [28].

The most commonly used animal species is the mouse. This is mainly due to the many advantages these animals offer. Many of these reasons are of a more trivial nature, which, however, means real differences in the feasibility of many studies in everyday laboratory life. The high reproduction rate is an essential component for the use of mice in research and science. This allows costs to be kept low and genetic manipulations and experimental approaches to be carried out and tested quickly and efficiently. Another essential component is the aforementioned genetic manipulation capability or the widespread and established expertise in interfering with the genetic material of mice in order to be able to specifically answer or simulate specific problems and disease patterns. However, mice are by nature relatively well protected against the development of atherosclerosis as they have a very high proportion of high-density lipoproteins in their blood, and genetic manipulation is therefore unavoidable [27]. However, as described above for rabbits, mice show a preferential localization of atherosclerosis formation in the thoracic region, whereas in humans − in relation to the aorta − infrarenal formation is predominant (shown in Fig. 2).

Especially with regard to the investigation of atherosclerotic vascular changes, which represent a chronic clinical scenario and thus take a lot of time to develop, it is advantageous to use an animal species such as the mouse in order to quickly obtain corresponding results. In the run-up to a study, however, one must also be aware of the disadvantages of a mouse model. For example, the technical feasibility of surgical interventions and the reproducibility of the experimental results can only be achieved with trained staff. The size ratios in the mouse model require microsurgical knowledge as well as the necessary routine to perpetuate validity in more complex procedures, e.g., on the aorta, after reaching a good experimental level with a preceding learning curve [29]. In the past, apolipoprotein E-deficient mice (ApoE−/−) and LDL receptor knockout animals (LDLr−/−) have been the most popular animal models for atherosclerosis research, although there is also a combination of both manipulations as a so-called double knockout variant (shown in Table 1).

- Genetic manipulations significantly increase the potential of animal models

In the ApoE−/− mouse model, all possible forms of atherosclerosis can be observed and even accelerated by special diets. With such profound genetic manipulations, however, it is not excluded that other regulatory circuits are also affected, such as the immune system, inflammatory cascades, and the behaviour of macrophages and smooth muscle cells, which also depend on an ApoE interaction. Moreover, this model mainly concerns the VLDL fraction, whereas in humans, it is mainly the LDL fraction that is important in the development of atherosclerosis. The biggest problem, however, remains that although atheromatous plaques form in these mice, these plaques have less clinical relevance than in humans since in mice these plaques do not tend to rupture with subsequent thrombosis of the vessel [30]. A similar type of cholesterol elevation in plasma is followed by the abovementioned deactivation of the LDL receptor, which takes up the cholesterol-rich LDL into cells by endocytosis. This lack of uptake of LDL results in mild hypercholesterolaemia under normal feeding conditions, which is greatly altered when an appropriately fatty feed source is given. This alternative shows greater homology to humans in terms of plasma composition as it has less effect on VLDL but more on the LDL fraction and also has no effect on the inflammatory cascade [27].

Other common animal models include ApoE3-Leiden and gain-of-function mutant pro-protein convertase subtilisin/kexin type 9 − adeno-associated virus (PCSK9-AAV8 or AAV9) mice, which should not be mistaken for the previously mentioned ApoE-deficient rodents. ApoE3-Leiden animals show a less pronounced tendency to develop atherosclerosis than ApoE−/− variants but have other advantages that come into play. Under an appropriate diet, the plasma levels of these animals show dramatically increased cholesterol and triglyceride levels, mainly due to the VLDL/LDL particles. Thus, these animals show a more human-like expression pattern of lipoproteins than other genetic variants.

- Rodents have a different lipid profile than humans

Furthermore, the production of ApoE is not reduced in these animals, which is why it is possible to study the effects of the disease without confounding inflammatory variables [31]. Even under normal feeding conditions, foam cell accumulations form early, which under an appropriate diet can lead to the formation of plaques along the entire aorta and its main trunks (including the coronary arteries) into the iliac vessels within a few months [32].

Gain-of-function mutant PCSK9-AAV8 or AAV9 mice represent animal strains whose genetic modification is carried out without germline intervention and is described as a cost-effective and rapid model for atherosclerosis research. A protease is produced that interacts with the LDL receptor on the cell surface and leads to its degradation, which is why the plasma LDL can no longer be taken up by cells. Already after a single i.v. administration of the virus, a stable expression of the protease could be detected without observing liver damage or immunological reactions [33]. The administration of diets rich in cholesterol resulted in significantly increased plasma cholesterol levels, with an equal distribution of VLDL and LDL fractions, which ultimately led to pronounced atherosclerosis formation [34].

## Summary

A basic requirement for an animal model to study atherosclerosis is the possibility of inducing a specific lesion within a certain time frame. In order to improve translatability to humans, several animal models are needed, which must be selected according to the research question. These range from large animals such as pigs to small animal experiments with genetically modified rodents such as the ApoE−/− mouse. In this way, animal models have made a decisive contribution to understanding the mechanism of origin and pathophysiology in the development of atherosclerosis and thus to evaluating targets for therapeutic options.

## Conclusion

- There is no “ single ” animal model for the study of atherosclerosis.
- Animal models are suitable for investigating intermediate stages of atheroma development.
- Advantages of large animal experiments such as human-like physiology and anatomy come at the expense of costs and handling.
- Genetic manipulation of small animal models opens up the possibility of addressing a wide range of questions.
- Genetic manipulations, but also “simple” feeding experiments can have massive, undesirable changes in inflammatory reactivity.
- In the future, elegant methods such as gain-of-function mutant PCSK9-AAV8 or AAV9 mice without germline intervention could open up new possibilities.

## Conflict of Interest Statement

The authors have no conflicts of interest to declare.

## Funding Sources

The authors have no fundings to declare.

## Author Contributions

All the authors, Florian Simon, Axel Larena-Avellaneda, and Sabine Wipper, drafted, wrote and critically revised the article together.

## Figures and Tables

**Table 1:** Overview of the lipoprofiles of individual species

	LDL	Human-like lipoprofile	HDL	VLDL	Triglycerides
Swine	+++	+++			
Rabbit					
WHHL	+++	+++			
NZW	+++	+++			
ApoE^−/−^	+++	+++			
Rat					
Wild-type	−––		+++	−––	
Diet					+++
Triton				+++	+++
Mouse					
Wild-type		−––	+++		
ApoE^−/−^	+	−––	−––	+++	
LDLr^−/−^	+++	+	−––	+	
Double knockout	+++	+++		+++	
ApoE3-Leiden	+	−––	+	+++	+++
Gain-of-function mutant PCSK9-AAV8	+	+	−––	+	

+++ (strong increase), + (slight increase), −−− (decrease or constant level). WHHL, Watanabe heritable hyper-lipidaemic; NZW, New Zealand White; HDL, high-density lipoprotein.
